# Phenotypes of Bronchopulmonary Dysplasia

**DOI:** 10.3390/ijms21176112

**Published:** 2020-08-25

**Authors:** Shih-Hsin Wang, Po-Nien Tsao

**Affiliations:** 1 Department of Pediatrics, Far Eastern Memorial Hospital, New Taipei City 22060, Taiwan; clara.wsh@gmail.com; 2 Department of Pediatrics, National Taiwan University Hospital, College of Medicine, National Taiwan University, Taipei 100225, Taiwan; 3 Center for Developmental Biology & Regenerative Medicine, National Taiwan University, Taipei 100226, Taiwan

**Keywords:** bronchopulmonary dysplasia, metabolomics, preterm, corticosteroids, phenotype, hyperoxia, inflammation

## Abstract

Bronchopulmonary dysplasia (BPD) is the most common chronic morbidity in preterm infants. In the absence of effective interventions, BPD is currently a major therapeutic challenge. Several risk factors are known for this multifactorial disease that results in disrupted lung development. Inflammation plays an important role and leads to persistent airway and pulmonary vascular disease. Since corticosteroids are potent anti-inflammatory agents, postnatal corticosteroids have been used widely for BPD prevention and treatment. However, the clinical responses vary to a great degree across individuals, and steroid-related complications remain major concerns. Emerging studies on the molecular mechanism of lung alveolarization during inflammatory stress will elucidate the complicated pathway and help discover novel therapeutic targets. Moreover, with the advances in metabolomics, there are new opportunities to identify biomarkers for early diagnosis and prognosis prediction of BPD. Pharmacometabolomics is another novel field aiming to identify the metabolomic changes before and after a specific drug treatment. Through this “metabolic signature,” a more precise treatment may be developed, thereby avoiding unnecessary drug exposure in non-responders. In the future, more clinical, genetic, and translational studies would be required to improve the classification of BPD phenotypes and achieve individualized care to enhance the respiratory outcomes in preterm infants.

## 1. Introduction

In recent decades, owing to the advancements in obstetrical and neonatal care, such as antenatal corticosteroid [1] and exogenous surfactant therapy [2], the survival rate of premature infants has improved dramatically. The incidence of many prematurity-related complications, such as respiratory distress syndrome (RDS), intraventricular hemorrhage, and necrotizing enterocolitis (NEC), has shown a significant reduction. However, bronchopulmonary dysplasia (BPD) is still one of the most common complications in preterm infants [3,4]. Approximately 40–45% of extremely preterm neonates develop BPD [4,5]. This disease leads to persistent pulmonary dysfunction, such as chronic wheezing, frequent respiratory tract infection, and exercise intolerance in young adults [6].

**Figure 1:**
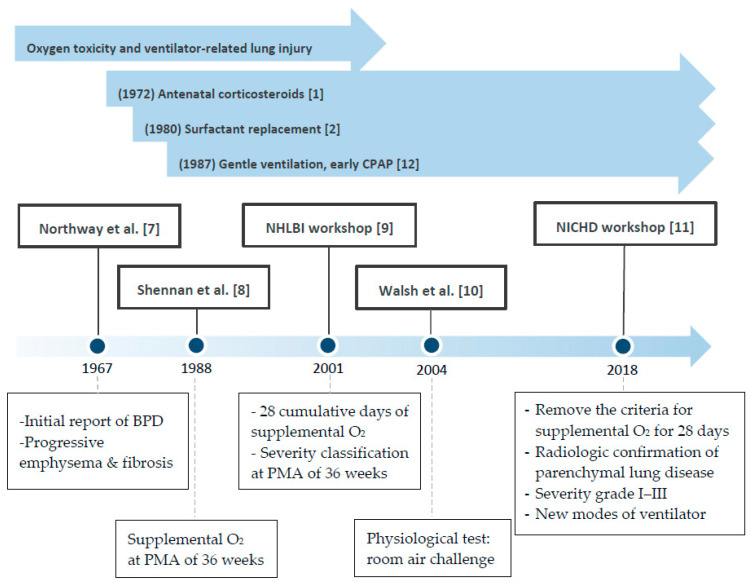
Bronchopulmonary dysplasia (BPD) definition and evolution. PMA: postmenstrual age. NHLBI: National heart, Lung, and Blood Institute.NICHD: National Institute of Child Health and Human Development. Pediatrics [1]. Lancet [2]. N. Engl. J. Med. [7]. Pediatrics [8]. Am. J. Respir. Crit. Care Med. [9]. Pediatrics [10]. J. Pediatr [11]. Pediatrics [12].

Both the definition and characteristics of BPD have evolved over the past 50 years (Figure 1) [7,8,9,10,11]. In 1967, when BPD was first reported by Northway [7], the clinical picture for “classic BPD” was severe fibrosis. However, in the era of routine application of antenatal steroid, surfactant, and gentler mechanical ventilation strategy [12] in the neonatal intensive care unit (NICU), the “new BPD” was proposed in 2000 [9]. The definition of BPD is more comprehensive and based on the need for supplemental oxygen for at least 28 days and further categorization of the severity into none, mild, moderate, or severe according to O_2_ requirement at the postmenstrual age (PMA) of 36 weeks [9]. In terms of the pathogenesis, the concept of maturation arrest of alveologenesis and angiogenesis, further leading to more diffused inflammation and less scarring and fibrosis, has been focused on. [13]. This definition of BPD is a practical reference in clinical settings but does not provide much information on longer-term respiratory outcomes and does not consider BPD as a multifactorial disease. Moreover, new respiratory strategies that use a nasal cannula with different degrees of oxygen make some infants unclassifiable per the current definitions. The previous criteria also do not include infants who die of respiratory failure before the PMA of 36 weeks and the time point of BPD diagnosis, which is the most severe form of lethal BPD. To address these knowledge gaps, the workshop on BPD held by the National Institute of Child Health and Human Development (NICHD) in 2016 refined the definition of BPD according to the previous criteria and present clinical care [11]. An infant born at less than 32 weeks of gestation; having radiographic-confirmed persistent parenchymal lung disease; and at 36 weeks PMA requiring ventilator support for more than three consecutive days, either invasive or non-invasive (N-CPAP, NIPPV, nasal cannula, O_2_ hood), to maintain arterial oxygen saturation above 90%, would be diagnosed with BPD. This refinement considers newer modes of non-invasive respiratory support and uses grades I, II, and III instead of mild, moderate, or severe. Grade III would be the most severe form of BPD and III(A) is a new category that covers the early-death group (patients who die between 14 days of postnatal age and PMA of 36 weeks because of parenchymal lung disease and respiratory failure), which has been overlooked for a long time [11]. A multicenter assessment of 2677 preterm infants [14] has indicated that BPD severity categorization based on the mode of ventilatory support at 36 weeks of PMA best predicts early childhood morbidity, regardless of supplemental oxygen use.

BPD is a complex disease with poorly characterized classification; this is one of the reasons why definite treatments are still lacking. Multiple factors contribute to the pathogenesis of BPD, which include developmental arrest of the premature lung, infection, oxygen toxicity, and ventilation-induced lung injury, making it difficult to classify the disease into subgroups or the so-called clinical phenotypes. Different phenotypes are associated with different outcomes. Therefore, understanding the diversity of phenotypes may enable better risk stratification and formulation of targeted treatment guidelines [15,16]. Metabolomics is a promising field that involves study of the associations between changes in the metabolic pathways and human diseases, and may help uncover the underlying mechanisms of BPD as well as facilitate the identification of useful biomarkers in early diagnosis, treatment selection, and prognostic prediction [17,18].

Chronic inflammation and hyperoxia have been considered as the important mechanisms for BPD and a common pathway leads to different phenotypes. Corticosteroids have been commonly used to prevent or treat established BPD. However, there are debates about the right kind of steroid, and the right dosage with the right route at the right timing to get the most effective result and simultaneously attenuate the negative impact. Using steroids in preterm infants is challenging because of the variability in clinical effects and the inability to target the possible responders prior to the treatment [19]. Pharmacometabolomics may provide a solution to this clinical dilemma. By measuring the metabolites along with the treatment course, the drug response may be quantified more clearly.

In our review, we focus on the pathology-driven nomenclature of the multiple clinical phenotypes of BPD, updated research regarding the inflammatory pathway and biomarkers for early prediction of high-risk infants prone to developing BPD, and early initiation of preventive strategies. The recent scientific advances in the field of metabolomics and pharmacometabolomics also offer new opportunities to approach the complex pathophysiology of BPD. The ultimate goal would be to develop comprehensive and personalized care for these individuals to combat this devastating disease.

## 2. Respiratory Phenotypes

Based on the widely accepted diagnostic criteria, BPD is classified into mild, moderate, or severe types according to the respiratory support or the need for supplemental oxygen at PMA of 36 weeks for preterm infants [9]. However, this classification does not adequately represent the heterogeneous pathophysiology in an individual infant. BPD is now considered a clinical syndrome characterized by different mechanisms and involves different regions such as airways, alveoli, and vascular components that may have different impacts throughout life [10].

Wu et al. [15] performed a retrospective, single-center study of a referral cohort of preterm infants with severe BPD, aiming to define the frequency of three disease components and the clinical outcome at hospital discharge. A total of 73 of the 76 (96%) evaluated infants were classifiable and belonged to one or more of the phenotypic subgroups: up to 78% had parenchymal lung disease, 66% had pulmonary hypertension (PH), and 60% had large airway disease. The most common phenotypic presentation was the co-occurrence of all three disease components. PH and large airway disease, instead of moderate-severe parenchymal disease, stand a higher chance of having a worse composite outcome, including death prior to NICU discharge, tracheostomy, or pulmonary vasodilator use when discharged [15]. PH alone was associated with an increased risk of mortality [15]. Next, we will discuss the different phenotypes and the corresponding current treatments of BPD (Table 1).

### 2.1. Central Airways

Tracheomalacia, subglottic stenosis, bronchomalacia, and bronchial stenosis are the clinical manifestations of central airway diseases commonly seen in BPD [29]. The positive pressure ventilation may damage the highly susceptible preterm airway, leading to the development of malacia [20]. The prevalence of large airway malacia, such as tracheomalacia or bronchomalacia, was reported to be less than 2% among extremely preterm infants [21]. However, this prevalence may be underestimated because of the unawareness, lack of diagnostic criteria [36], and limited availability of bronchoscopic examination [37], and because the procedure was performed only in a small number of preterm infants [16,38]. To maintain a patent airway, infants with large airway disease were associated with a higher need for tracheostomy and home ventilation [15,21]. The symptoms of tracheomalacia usually improve gradually and resolve by age 2 [36]; however, the lifelong impact is not clearly known [16].

### 2.2. Small Airways

The small airways component of BPD, consisting of structural remodeling, bronchoconstriction, and hyperreactivity, manifests similarly to asthma [16,29]. Clinically, it may present with airway obstruction. The obstructive lung disease seen in BPD includes both a fixed component secondary to structural changes that respond poorly to standard asthma therapies and a reactive inflammatory component that responds fairly [16]. A study found that the survivors of BPD at school age present with low exhaled nitric oxide levels and poor response to β2-agonists during the evaluation for airflow limitation, which is quite different from the conditions of other typical asthmatic children. These data indicate that the pathogenesis of obstructive lung disease in patients with BPD might involve the structural changes in small airways instead of airway inflammation [39]. Of course, besides airway structural changes, airway inflammation also contributes to the development of BPD [40,41]. Recent studies have demonstrated that BPD is associated with a certain cytokine expression pattern, which suggests these cytokine profiles might contribute to the development of BPD in preterm infants [42]. However, it is unclear whether this cytokine profile was triggered by postnatal infection or it pre-existed in some preterm infants prenatally [43]. For example, maternal ureaplasma chorioamnionitis could trigger a pro-inflammatory state, which may contribute to the pathogenesis of developing BPD [44]. Studies in mice, rats, and rabbits have shown that blockers of inflammatory mediators, receptors, and signaling pathways improve BPD presentation [45]. However, using inflammatory modulators in treating BPD in infants has not been uniformly successful [46,47]. It is also unclear whether interventions such as corticosteroids that modify the inflammatory state alter long-term respiratory outcomes [22].

### 2.3. Distal Airspace and Vasculature

The “new” BPD focuses more on the aberrations in both alveolar and pulmonary vascular development and the distal component comprises decreased alveolarization, abnormal vascular remodeling, and impaired lymphatic function. The alveolar disease component results in impairment of gas exchange including poor oxygenation and hypercapnea [16]. The interruption of alveolarization by preterm birth leads to the development of fewer but larger alveoli and presents with reduced vital capacity, confirmed by lower carbon monoxide diffusion in BPD infants around the corrected age of 17.4 months [16,48]. Fortunately, owing to ongoing alveolar multiplication during postnatal life, most patients with BPD can be weaned off from ventilator support successfully [27,49,50,51,52]. However, many children and young adults with a history of preterm birth still present with exercise limitation and reduced pulmonary reserve, even with normal lung capacity [53,54,55].

Researchers nowadays pay more attention to the effects of prematurity on vascular development and focus more on pulmonary vascular disease in BPD [56]. The distal airspace has a complex interaction with the surrounding vasculature during lung growth and thus generates the concept of ‘vascular hypothesis’ of BPD, highlighting the contribution of the impaired lung vascular growth to the disordered development of distal airspace in the setting of preterm birth [57,58,59,60]. Pulmonary vascular disease (PVD), the vascular component of BPD, encompasses a spectrum of PVD phenotypes. Among them, PH has long been recognized as a strong contributor to poor survival in preterm infants with BPD [61]. However, the PVD spectrum is too wide, making it a challenge to define the presence of PH and the quantification of PVD under universally consented diagnostic standards [62]. This would be especially difficult in infants with “subclinical” PVD [56]. During intrauterine life, PH is necessary to support fetal circulation since the placenta is the main organ for gas exchange. Immediately after birth, a rapid and dramatic decrease in pulmonary vascular resistance occurs to direct the blood flow into the lungs of the newborn allowing for further ventilation and perfusion adaptation. Under certain circumstances, such as severe parenchymal lung disease, hypoxic-related intra-uterine growth restriction, oligohydramnios, prolonged premature rupture of membranes, or genetic predisposition [63,64,65,66], the vascular transition is delayed, and the pulmonary vascular resistance (PVR) does not decrease normally. It leads to extrapulmonary right-to-left shunting through the ductus arteriosus or across the foramen ovale, further resulting in profound cyanosis and hypoxemia [63,64,65]. Of note, the earlier we confirm the delayed pulmonary vascular transition by echocardiography [65], the higher the risk of developing BPD and pulmonary hypertension [65,67]. Based on retrospective studies, PH may occur in up to 17–43% of BPD affected preterm infants [68,69,70,71], and the mortality rate may be as high as 14–38% [61]. The affected infants often present with recurrent cyanotic episodes and have prolonged initial hospital admissions, require extra oxygen supplement or higher levels of respiratory support even after discharge compared with infants with BPD alone [72]. In terms of treatment strategy, the commonly used medications are the pulmonary vasodilators that deal with the reactive component of PH. In contrast, for the fixed component secondary to an underdeveloped pulmonary vascular bed, there is currently little that clinicians can do [16,25]. In the long run, children with chronic pulmonary vascular disease might have different degrees of exercise intolerance and persistent echocardiographic abnormalities along with cardiac remodeling and structural change into young adults [29]. Even among preterm infants of similar gestational ages, extreme phenotypic and severity variability in PVD exists and reflects in variable responses to vasodilators. Thus, researchers are trying to identify biomarkers for following PH longitudinally into further childhood [73].

## 3. History and Rationale for Using Postnatal Corticosteroids (PCS)

BPD is now considered a multifactorial disease and both prenatal and postnatal risk factors play a role in this disease. Exposure to an inflammatory environment has been one of the well-accepted theories regarding the development of BPD [74,75,76,77,78]. The exposure may begin prenatally. Preterm infants born to mothers with chorioamnionitis present with evident lung inflammation during postnatal days and are more prone to developing BPD [79]. Ureaplasma species, frequently colonized in the lower genitourinary tract of women, can invade the amniotic fluid to cause inflammation, making it the most common organism associated with chorioamnionitis. Antenatal exposure to *Ureaplasma* spp. induces pulmonary inflammation in preterm sheep, and evidence from other experimental data demonstrate a significant association between ureaplasma chorioamnionitis and BPD development in preterm infants [44]. Furthermore, intra-amniotic administration of potent pro-inflammatory stimuli, such as lipopolysaccharide (LPS) or *Escherichia coli* endotoxin in animal models seems to be harmful to the normal alveolar and vascular growth [80,81].

After birth, in addition to developmental arrest of the immature lung, the preterm infants are continuously exposed to inflammatory environments, including lung and systemic infection owing to immature immunity, invasive procedures, oxidative stress and free radicals generated from hyperoxia, and mechanical ventilation. The incidence of BPD increased among very-low birth-weight infants with a history of either early- or late-onset of sepsis [82,83]. By using newer and gentler ventilation methods, ventilator-associated lung injury has diminished, but inflammation remains the key basis of pathogenesis [84]. Antenatal steroid therapy reduces mortality and many prematurity-related morbidities, including RDS, intraventricular hemorrhage, and necrotizing enterocolitis, but BPD remains unsolved [85].

Since corticosteroids are powerful downregulators of inflammation, there is a long history of using corticosteroids postnatally to prevent and treat established BPD. Physiologically, steroid hormones are produced in the adrenal cortex. There are two main classes of corticosteroids—glucocorticoids and mineralocorticoids—involved in a wide range of metabolisms and the immune system during physiological stress. Cortisol, an endogenous glucocorticoid, is released in response to stress, and it aids in the metabolism of carbohydrate, fat, and protein, to increase blood sugar level and blood pressure, to control inflammation, and to suppress the immune system. However, fetal cortisol production only reaches significant levels after 30 weeks of gestation, and before 23 weeks of gestation, the production is very limited because of the immature hypothalamic-pituitary-adrenal axis [86,87,88]. As a result, the capacities of these extremely preterm infants to handle extrauterine stress, such as mechanical ventilation, painful procedures, infection, or noxious stimuli, are impaired. The imbalance between the immature adrenal gland function and the ongoing inflammatory process in preterm neonates has prompted studies investigating the effect of corticosteroids on the development of BPD for the past three decades.

There has been a long debate on the use of PCS. In the early 2000s, concerns about serious short- and long-term side effects such as neurodevelopmental impairment outweighed the possible beneficial effects of prevention or treatment of chronic lung disease, leading to recommendations against the routine use of systemic dexamethasone [89]. A small randomized controlled trial using low initial and total doses of dexamethasone after the first one week of life revealed shorter ventilator-dependent duration in preterm infants without obvious short-term morbidities [90], and there was no strong association with long-term complications [91]. This study reopened the debate regarding the use of corticosteroid therapy, especially in infants with multiple risk factors of BPD. Since both severe BPD and PCS are associated with impaired neurodevelopment, further evidence suggested that clinicians should balance the risks and benefits more carefully and consider the use of PCS when the risk of BPD is high (>50%) [92]. A European cohort in 2011 showed that PCS were prescribed for 13.9% of infants born between 24 and 29 weeks of gestation but the use of PCS in clinical practice varies widely across regions (3.1–49.4%) [93]. Some neonatal factors are associated with PCS prescription including low gestational age, small for gestational age, male sex, under mechanical ventilation, or receiving non-steroid anti-inflammatory drugs for patent ductus arteriosus (PDA). It may partly suggest that disease severity plays a role in, but cannot fully explain, the variation in PCS use. Despite all the concerns, the use of PCS has never been abandoned and further evaluation of the different kinds of corticosteroids and routes of administration may help to get the most out of steroid therapy [29].

### 3.1. Dexamethasone

Dexamethasone has been used in various studies owing to its two main characteristics, its potent glucocorticoid activity and long half-life [94]. Avery et al. published a controlled trial of dexamethasone in ventilator-dependent infants in 1985 and showed short-term improvement in lung function, especially a striking increase in compliance within 48–72 h after treatment, permitting rapid weaning from the ventilator [95]. To date, the debate on the use of dexamethasone is still ongoing. Recent Cochrane reviews by Doyle concluded that the benefit of corticosteroids, particularly dexamethasone, either given early (≤7 days) or late (>7 days), may not outweigh the adverse effects associated with this treatment [96,97]. Even though early corticosteroid treatment can improve pulmonary function rapidly, facilitate extubation, and reduce the risk of BPD and PDA, there are concerns, including acute complications, infection, gastrointestinal (GI) perforation, and also longer-term sequels such as growth inhibition and central nervous system (CNS) injury. Dexamethasone interferes with the hypothalamic-pituitary-adrenal axis, suppresses endogenous cortisol production, binds only to glucocorticoid receptors, and leads to apoptosis in the hippocampus [98]. Current consensus reserves the use of late corticosteroids for patients who are difficult to wean from the mechanical ventilator and to minimize both the dose and duration of any course of treatment [97].

### 3.2. Hydrocortisone

Since dexamethasone has a strong affinity for only the glucocorticoid receptor and long half-life, its clinical shortcomings are apparent. Researchers have turned to different kinds of PCS with different routes of administration. Preterm infants with lower cortisol levels in the first week of life have been linked to prolonged oxygen dependence and the development of BPD, possibly because of their inability to secrete adequate amounts of cortisol in response to stressful stimuli, resulting in damage to the vulnerable lung [99]. In addition, infants presenting with symptoms and signs of hypotension are sometimes treated with a stress dose of steroids. Thus, an individual patient data meta-analysis, including five eligible studies in extremely preterm infants, evaluating the efficacy of using early low-dose hydrocortisone as prophylaxis of adrenal insufficiency demonstrated that the therapy is beneficial for survival without BPD [100]. The most updated results for the use of hydrocortisone came from the PREMILOC trial [101]. They found that the rate of BPD-free survival at 36 weeks of PMA was increased significantly by prophylactic low-dose hydrocortisone (0.5 mg/kg every 12 h for 7 days and then 0.5 mg/kg daily for 3 days) [101]. Compared to a previous study that was stopped prematurely because of the increase in the frequency of GI perforation, the PREMILOC trial showed no increase in GI or other short-term complications, and the long-term neurodevelopmental outcomes were also similar, or even improved in extremely preterm infants [102,103]. In addition to the clinical prognosis, the PREMILOC study also evaluated the hydrocortisone effect on brain structure by assessing brain MRI at term equivalent of age and concluded that neither white matter brain damage nor overall moderate-to-severe brain lesions were statistically different among those who had early hydrocortisone exposure when adjusted for other neonatal variables [104]. The exposed infants should have a neurodevelopment assessment at preschool age to verify the safety of early hydrocortisone because re-evaluation at this age reflects a child’s general intellectual ability more accurately [105].

Regarding the timing for hydrocortisone intervention, a recent meta-analysis involving 12 studies using early (within the first week of life) and two using late hydrocortisone demonstrated that early systemic use significantly alleviated BPD at PMA of 36 weeks and also attenuated neurodevelopmental impairment. Some safety concerns remain, owing to a higher risk of intestinal perforation, especially among patients under NSAIDs treatment for patent ductus arteriosus at the same time. Owing to the limited number of studies using late hydrocortisone, there is no strong conclusion currently [106].

### 3.3. Inhaled Corticosteroids

Inhaled corticosteroids have been postulated to be an alternative strategy to decrease systemic absorption and the side effects. Owing to its strong topical effects, the incidence of BPD was lower among infants receiving early inhaled budesonide [107] but the effective delivery method for inhaled glucocorticoids in preterm infants is still under investigation.

In 2017, Shah et al. [108] presented this challenge in a Cochrane review, which found no net advantages of inhaled corticosteroids over systemic corticosteroids, neither in the primary outcome with the incidence of death or BPD at 36 weeks of PMA nor in the secondary outcomes as ventilator-dependent duration or length of admission course. In contrast, the adverse event profiles for inhaled compared with systemic steroids were similar. These results urge researchers to design a better delivery system for the inhaled steroids, ensuring selective delivery to the alveoli [108]. From animal studies, we have learned that aerosolized budesonide mostly remains in the airway, but with the help of surfactant, it distributes more evenly into distal alveoli, enhancing gas exchange and lowering lung inflammation [109,110,111,112,113]. In a meta-analysis involving two clinical trials [114], intra-tracheal administration of budesonide combined with surfactant at birth reduced BPD by 40% without a difference in mortality or other well-known adverse outcomes [41,115]. However, there are still concerns about using an inhaled corticosteroid in preterm infants because of their immature drug metabolic system. In children and adults, budesonide is kept in the lung tissue as budesonide esters for delayed glucocorticoid release, but in preterm animal models, this pharmacokinetic feature has not been definitely demonstrated [116,117,118,119]. The CYP3A enzymes in the liver metabolize and inactivate budesonide, which is absorbed systemically, further minimizing systemic exposures. However, these enzymes may be less developed in premature infants [119,120,121]. Studies in preterm sheep demonstrated that budesonide 0.25 mg/kg with surfactant is absorbed into the systemic circulation and alters hundreds of genes in the liver and brain [122]. A previous study also showed that the affinity of budesonide for the glucocorticoid receptor is about 10 times higher than that of dexamethasone [123]. The recent data on preterm lambs from a study by Hillman et al. searching for the lowest effective dose of budesonide for decreasing lung injury is consistent with the dose Yeh et al. used in their clinical studies, 0.25 mg/kg, though systemic effects should be monitored in infants [119]. Currently, a multicenter randomized controlled trial (Preventing Lung Disease Using Surfactant + Steroid, PLUSS) is ongoing to test whether early administration of intratracheal budesonide combined with surfactant (Curosurf) reduce the rate of BPD or death at 36 weeks of PMA compared with Curosurf alone. The study is recruiting 1060 extremely preterm infants, less than 28 weeks of gestation, born in 2017–2022 from centers in Australia, New Zealand, and Canada. It may provide more evidence for clinical practice.

To obtain the best clinical outcome, we need to know what kind of steroid to use with the right dose at the right time through the right route for the appropriate duration. Future studies should focus on the pathobiology at the molecular level; looking for practical biomarkers and the genetic-environment interplay will help to design clinical trials and elucidate the phenotypic responders.

## 4. Recent Advances in Hyperoxia Exposure and Inflammatory Pathway of BPD

The incidence of BPD remained the same [124,125,126] or even higher because the smallest preterm infants survived [5,127]. The most affected population is infants born less than 28 weeks of gestation [4,13,128,129]. Along with a population shift, the key pathological hallmark of BPD today is a changing histopathological picture with disrupted development of the alveolar airspaces of the lung [13,129]. There are several mediators impairing alveolarization that recent studies have focused on, including the oxidative stress-related genes, TGF-β signal pathway, inflammatory pathways, extracellular matrix modification or dysregulation, and non-coding RNA [130]. Among these, lung inflammation remains a key factor in the aberrated alveolarization of BPD; however, more studies are needed to understand the molecular mechanisms underlying the injurious effects on lung growth and the endogenous pathways invoked to preserve lung development during inflammation [131]. The inflammatory response is believed to be triggered antenatally by intrauterine stress or chorioamnionitis-related cytokine exposure and further increased postnatally by factors such as hyperoxia, ventilator-induced lung injury, systemic infections, or inadequate nutritional support [132]. It also explains why researchers use hyperoxia-exposed animal models to study the antioxidant responses or to understand lung development under oxidative stress. Immediately after birth, the infants are exposed to an oxygen-rich environment compared with the intrauterine life. For the extremely preterm babies, they often require even higher oxygen supplement during resuscitation and initial stabilization. Oxygen is a double-edged sword since the preterm infants do not have an adequate amount of endogenous antioxidants to deal with oxidant injury, further predisposing them to the development of BPD or NEC [133,134]. The influences on cell growth brought by hyperoxia are either from the accumulation of inflammatory mediators or direct insult from reactive oxygen species [135]. These reactive species cause damage to not only cellular macromolecules [136,137], but also at the nuclear or mitochondrial DNA level. Quantitative analysis of DNA demonstrated that both DNA strands broke and base damage occurred in the lungs of mice in a hyperoxia environment [138]. DNA strand breakage, despite a seemingly secondary effect during cell death in oxygen, occurs mainly in alveolar type 2 cells and relates to lung injury [138]. In addition to the direct DNA damage, epigenetic changes, such as acetylation of histone, also affects cell cycle regulation and are associated with hyperoxia-induced alveolar simplification [139]. Other cells in the lung are also affected by hyperoxia. The lung epithelium presented with markedly increased apoptotic cell numbers in the hyperoxia group and can be protected by Calcitonin gene-related peptide [135]. Fibroblasts help maintain alveolar and bronchial integrity, but following hyperoxia, significant morphological changes occur, with decreased proliferation and increased cell size, leading to a pro-fibrotic stage and resulting in abnormal repair in the lung [140].

The effect of antioxidants was demonstrated in a preclinical study of guinea pigs receiving glutathione additive parental nutrition; it seemed to have rescued the oxygen-related injury and prevented the loss of alveoli [141]. Certain genetic variants of antioxidant response genes, such as the single nucleotide polymorphism in the NFE2L2 gene or NQO1 gene, altered the susceptibility to BPD or the disease severity in preterm infants [142,143,144]. These effects were further examined using pharmacological induction of gene expression, but with variable results. For instance, the pharmacological induction of *Nfe2l2* expression did not reverse the developmental arrest of alveolar growth after hyperoxia [145]. However, for the NQO1 gene, it increased genetic expression pharmacologically in mice and rescued hyperoxic lung injury [146]. The development of airways and pulmonary vasculature is a highly coordinated process during lung growth, and oxidative stress disturbs the interaction among them. The cGMP signaling is one of the key pathways regulating pulmonary vascular tone in neonates. After hyperoxia exposure, it also plays an important role in the pathogenesis of PH phenotype in BPD patients [147]. Superoxide dismutase (SOD) is a class of antioxidant enzymes that catalyzes the dismutation of the superoxide radical into oxygen and hydrogen peroxide. There are three different SOD isoforms in mammalian cells. Previous animal studies have found that different isoforms have different degrees of protection against hyperoxia-induced damage. SOD_3_ is highly expressed in the arterial wall and loss of SOD_3_ results in alveolar simplification, vascular remodeling, and pulmonary hypertension [148]. The studies above have emphasized the importance of optimal saturation targeting and broadened our understanding on the balance between oxidative stress and the lung antioxidant mechanism. These have also shed new light on the pathogenesis of BPD and might provide researchers with possible therapeutic targets in the future.

Several studies have focused on the molecular pathobiology involved in distal lung development during inflammatory stress. They found that the BPD preterm infants have higher levels of inflammatory cells in their tracheal aspirate following the elevation of IL-8 and IL-6 in the earliest phase of lung injury, which initiated a series of detrimental cascades and ended up in pulmonary microvascular edema, limiting epithelial growth factor expression and suppressing pulmonary development [149,150]. One of the cascades worthy of attention is the nuclear factor kappa B (NF-κB) signaling pathway. This NF-κB pathway is a key regulator in the release of cytokines and chemokines and can cause excessive inflammation resulting in lung diseases. Pharmacological inhibition of the NF-κB signaling pathway had an impact on lung alveolarization and pulmonary angiogenesis in newborn mouse pups [131]. The sophisticated interplay between the epithelium and the mesenchyme is crucial for normal lung development. Perinatal inflammation can damage the elastin assembly and blunt lung alveolarization by disrupting the NF-κB pathway in a mouse study [151]. The same study also demonstrated that the restoration of elastic fiber assembly in the developing lung is possible [151]. To test the possible targets for inflammatory modulation, several hyperoxia-based mice models have focused on the benefits of different interleukin (IL)-1 receptor antagonists, with or without combining other anti-inflammatory agents (such as protein C), to rescue the devastating effects on lung alveolarization and further examine other possible therapeutic targets, such as the Rac1 signaling pathway [152,153,154].

The adaptive immune system is also involved in inflammation-related lung pathology, probably because of their roles in tissue migration and immune surveillance. Several reports have demonstrated that T cell activation and alteration in lymphocyte subsets are related to several prematurity associated complications, such as BPD, NEC, and periventricular leukomalacia [42,155,156]. Umbilical cord blood collected from preterm infants present with lower levels of naïve CD8+ T cells and less degree of regulatory CD31 expression, which may explain why these small neonates are vulnerable to CD8+ T cell-mediated inflammation and have impaired T cell memory function [157]. Both chorioamnionitis and BPD are associated with pro-inflammatory conditions; therefore, it is not surprising that pro-inflammatory CD4+ non-regulatory T cell cytokines are increased. However, there is a decreased abundance of regulatory T-cells in the later disease, suggesting certain inflammatory changes in these chorioamnionitis-exposed and BPD infants [158]. In a preclinical study with a hyperoxia-based mouse model, anti-inflammatory CD11b+ mononuclear cells protected the developing lung from injury, suggesting that enhancing their functions may provide some benefits to prevent BPD [159].

Apart from the immune functions, trophic functions of macrophages are involved in many tissue development and physiological regulations as well [160,161]. Yolk sac-derived macrophages colonize the alveolar space and mature in the distal lung shortly after birth [162,163]. Myeloid-lineage macrophages are recruited to the lung under pathological conditions, including BPD, and provoke a localized inflammatory response. Macrophage activation inhibited the expression of multiple genes critical for physiological lung growth in vivo and reduced airway branching in vitro [164]. In assessing the causal roles of immune cells as mediators in lung organogenesis, it was found that a hyperoxia mouse model of BPD showed impaired lung recruitment of immune cells and that cells of the *Csf1r*-expressing monocyte/macrophage lineage contributed to stunted alveolarization [165]. Furthermore, hyperoxia-exposed animal models demonstrated a rapid accumulation of neutrophils in the airways right after the initiation of mechanical ventilation. The neutrophils become activated under postnatal inflammatory insults and adhere to the endothelium of the pulmonary vascular system, followed by decreased alveolarization and angiogenesis, thus leading to difficult consequences such as PH [78,166,167,168]. Administration of leukadherin-1 (LA1), a novel agonist of leucocyte surface integrins, enhances leukocyte adhesion to the vascular endothelium, prevents leukocyte transendothelial migration to the lung, and decreases macrophage influx to the injury site during hyperoxia exposure [169]. Other promising targets include the chemokine receptor 4 (CXCR4), which, together with the stromal-derived factor-1 (SDF-1), modulates the inflammatory response. Targeting CXCR4 with its antagonist alleviates lung injury with a decrease in macrophage and neutrophil counts in the bronchoalveolar lavage fluid [150].

The studies above highlight the role of inflammatory cells as pathogenic mediators and provide potential targets in the inflammatory signaling pathway to protect against injurious stimuli to lung alveolarization [130].

## 5. Screening and Prevention: Biomarkers and Omics

BPD is a multifactorial disease due to the exposure of an immature lung to complex interactions between genetic and environmental factors [170,171,172,173]. There are some commonly known risk factors for BPD, such as infection, hyperoxia, or ventilator-related lung injury, but not all exposed premature infants develop BPD [171]. Although there are methods to modify disease severity, there is no definite treatment and prevention. Early recognition of infants at risk and prevention using more personalized methods may be the only way to improve prognosis [174].

Although researchers are now working on BPD-related biomarkers, ranging from identifying infants at risk of disease development, early disease detection, understanding disease progression to effective and suitable intervention, to date, no single biomarker has been validated as clinically reliable. Metabolomics is an “omic” science involving the quantification and characterization of low-molecular-weight endogenous metabolites, reflecting instantaneous metabolic perturbation [175]. Considering the complicated mechanisms and different phenotypes of BPD, it would be more realistic to develop a panel of biomarkers, including those obtained using genomics, microbiomics, and metabolomics, to describe the metabolic profile. A recent review examined the evidence arising from metabolomics and its interaction with microbiomics and pharmacology [18]. The changes in metabolite composition represent the host response to a disease or during a pathophysiological state. Therefore, metabolomics can provide a unique fingerprint of a specific physiological interaction in an individual preterm infant.

Biomarkers can be identified in various body fluids, such as serum, cord blood, amniotic fluid, urine, tracheal aspirate, or bronchoalveolar lavage fluid. The metabolomics of BPD includes metabolites related to alterations in oxidative stress, lipid, and amino-acid metabolism. Baraldi et al. [176] used amniotic fluid to perform an untargeted metabolic analysis to investigate whether this approach could help identify infants prone to developing BPD. Twenty-one preterm neonates were analyzed according to their BPD status, and 10 of them developed BPD. Interestingly, the group with BPD was characterized by lower levels of 3β,16α-dihydroxyandrostenone sulfate, a metabolite of dehydroepiandrosterone sulfate. As mentioned earlier, one of the rationales for using corticosteroid is the observation of relative adrenal insufficiency in preterm infants. This metabolomic finding shows the association between developing BPD and adrenocortical insufficiency. This study suggests that the injury responsible for BPD may begin as early as intra-uterine life owing to prenatal oxidative stress exposure and metabolic dysregulations.

Pharmacometabolomics is a novel field of metabolomics aiming to identify changes in the metabolome before and after specific drug treatment. Analyzing the metabolomic change in conjunction with the drug response measurements and the clinical outcome will help scientists to elucidate the mechanisms of drug effect. Corticosteroids have shown promising effects in decreasing BPD but the unwanted side effects and highly heterogeneous drug responses pose an urgent need for a more individualized approach by identifying high-risk groups. Pharmacometabolomics may be a helpful tool to identify patients who might benefit from specific drug therapy [18]. Lewis et al. [19] performed the first pharmacometabolomic study in systemic-dexamethasone-treated BPD infants. They found that 11 metabolites in the blood and 15 in the urine differed significantly before and after steroid treatment. Among them, gluconic acid presented with the most significant change after steroid treatment, and the degree of urine gluconic acid change is related to drug response.

According to previous studies, gluconic acid, an oxidation product of glucose, has some distinct features that implicate inflammation. Fanos et al. [175] found that urine gluconates were associated with BPD development, taking four other urine metabolites into account. Another pilot study by Fattuoni et al. [177] also revealed that the only metabolite significantly upregulated in the cord blood of neonates born to mothers with chorioamnionitis was gluconic acid. Besides urine gluconic acid, the levels of uridine and mannitol also have a negative correlation with the degree of steroid response [19]. Conclusively, the changes in serum and urine metabolites indicate that dexamethasone therapy does have effects on the major metabolic pathways involved in the inflammatory cascade and oxidative stress [19].

Therefore, if we can identify and measure certain metabolomics biomarkers that correlate with drug response, or a “metabolic signature”, it may allow precise medical intervention with better patient selection in order to avoid exposure to the side effects without the benefits [18,19].

## 6. Conclusions

To reduce the complications associated with BPD, we need a multi-pronged and more personalized approach. First, timely recognition of both prenatal and postnatal risk factors for the development of BPD may enable primary prevention at the optimal timing for infants at the highest risk. Second, screening for predictive biomarkers for BPD may allow for earlier treatment within the window of opportunity. Third, understanding the pathophysiology of the preterm pulmonary development by recognizing dysregulated pathways in different phenotypes, identifying biomarkers, and testing novel or more patient-specific treatments may help improve outcomes. For instance, subtyping the inflammation in preterm respiratory disease more precisely may help guide clinical decision to select a possible responder to PCS [16]. Finally, identifying the serious BPD phenotypes, such as pulmonary hypertension, which causes clinical deterioration, may help develop treatment guidelines and reduce the burden of disease.

Research on validated biomarkers and the development of accurate, rapid, and affordable point-of-care biomarker tests has been both challenging and promising. A panel of biomarkers could be applied in combination with clinical parameters to serve as predictors for later disease or long-term prognosis. To modify clinical outcomes, the ideal biomarkers should be expressed early in the postnatal days to enable physicians offer timely and targeted interventions. Future directions will emphasize the classification of BPD phenotypes using biomarkers and novel therapies tailored to the underlying pathophysiology. Trials targeting certain phenotypic pathophysiology are more patient-specific and more efficient than the traditional randomized controlled trials that enroll all at-risk neonates [73]. Taking the benchwork to the bedside would lead to “precision medicine,” which will provide better care to the at-risk premature infants.

## Figures and Tables

**Table 1:** Phenotypes of BPD and current therapeutic practice.

Phenotypes		Current Therapeutic Practice
Central airways		Tracheostomy, home ventilator [20,21,22]
Small airways	Structural change	Poor response to standard asthma treatment [16,22]
	Airway inflammation	Bronchodilators [23,24] Systemic or inhaled corticosteroids [22,25] Leukotriene receptor antagonists [26]
Distal airspace and vasculature	Interruption of alveolarization	Postnatal alveolar multiplication [27]
	Pulmonary vascular disease	Inhaled Nitric Oxide [22,28,29] Oxygen therapy [25,29] Pulmonary vasodilators (Sildenafil, Bosentan) [25,30,31,32,33] Diuretics [22,34,35]

## Abbreviations

BPD	Bronchopulmonary Dysplasia
CLD	Chronic Lung Disease
PDA	Patent ductus arteriosus
PCS	Postnatal Corticosteroids
PMA	Postmenstral Age
